# Serial surveillance by circulating tumor DNA profiling after chimeric antigen receptor T therapy for the guidance of r/r diffuse large B cell lymphoma precise treatment

**DOI:** 10.7150/jca.60390

**Published:** 2021-07-13

**Authors:** Linghui Zhou, Houli Zhao, Yang Shao, Xin Chen, Ruimin Hong, Linqin Wang, Fang Ni, Arnon Nagler, Yongxian Hu, He Huang

**Affiliations:** 1 Bone Marrow Transplantation Center, the First Affiliated Hospital, Zhejiang University School of Medicine; 2 Institute of Hematology, Zhejiang University; 3 Zhejiang Province Engineering Laboratory for Stem Cell and Immunity Therapy; 4 Liangzhu Laboratory, Zhejiang University Medical Center, 1369 West Wenyi Road, Hangzhou 311121, China; 5 Nanjing Geneseeq Technology Inc., Nanjing, Jiangsu, China; 6 Chaim Sheba Medical Center, Tel Hashomer, Israel, Tel Hashomer, Israel

**Keywords:** ctDNA, DLBCL, CAR-T, precise treatment

## Abstract

**Background:** Circulating tumor DNA (ctDNA) released from tumor cells carries the tumor-associated genetic and epigenetic characteristics of cancer patients. Next-generation sequencing (NGS) facilitates the application of ctDNA profiling for identification and monitoring of minimal residual disease (MRD) in cancer, and can serve as the guidance for precise treatment.

**Methods:** In this study, we profiled genomic alterations in the baseline, relapsed, and progressive tumor samples of eight diffuse large B cell lymphoma (DLBCL) patients (NCT03118180) after chimeric antigen receptor T (CAR-T) cell therapy.

**Results:** The median follow-up was 41 months. 4 (50%) patients achieved complete remission (CR), 1 (12.5%) patient achieved partial remission (PR), and the other 3 (37.5%) patients showed no response. 3 of 5 patients who achieved remission relapsed within 4 months after CAR-T therapy, while the rest 2 patients remained CR for more than 3 years. Based on the positron emission tomography-computed tomography (PET-CT) scan, the current gold standard for evaluating response to therapy in lymphoma, the sensitivity and specificity of our ctDNA profiling in detecting tumor-related ctDNA mutations were 94.7% and 83.3%, respectively. The median numbers of baseline plasma ctDNA mutations in patients who remained long-term CR and patients who relapsed or became refractory to CAR-T therapy were 3 and 14.3, respectively. *GNA13*, *SOCS1*,* TNFAIP3* and* XPO1* mutations appeared to be associated with poor prognosis after CAR-T cell therapy. Our results also suggested that lenalidomide might relieve relapsed lymphoma with mutations in *NFKBIA* 202C>T (p.Q68*) and *NFKBIE* 433A>T (p.K145*) by targeting NF-Kappa B signaling. In addition, the inhibitor selinexor may be another choice for refractory or relapse (r/r) DLBCL patients after CAR-T cell treatment.

**Conclusion:** Serial ctDNA monitoring is an emerging technology for the surveillance of disease status and prognosis prediction. In this work, we demonstrated the use of serial ctDNA monitoring in r/r DLBCL patients after CD19-targeted CAR-T cell therapy. Our longitudinal NGS profiling revealed the changes of ctDNA mutation in accordance with prognosis, and shed some light on exploring more targeted treatment options together with CAR-T cell therapy.

## Introduction

DLBCL is the most common subtype of non-Hodgkin lymphoma 1. Although it is an aggressive lymphoma, DLBCL is considered curable with about 43.5% 10-year overall survival, especially with the addition of rituximab.^2^ However, approximately 30-40% of patients relapse or progress after rituximab 3. In fact, more than half of DLBCL patients still cannot reach the goal of long-term survival, often due to the high heterogeneity of DLBCL and the absence of effective treatment 2. Most DLBCL patients that relapse or become refractory to chemotherapy will die from progression disease 4.

Lymphocyte depletion followed by autologous CAR-T cell infusion has already shown promising effectiveness in treating relapsed or refractory (r/r) DLBCL 5-8. To date, five CAR-T therapies, including axicabtagene ciloleucel, tisagenlecleucel, brexucabtagene autoleucel, lisocabtagene maraleucel and idecabtagene vicleucel have been approved by the United States Food and Drug Administration (FDA) in October 2017, May 2018, July 2020, February 2021 and March 2021, respectively. CAR-T cell therapy is usually used as the last resort for the treatment of refractory and relapsed B cell lymphoma. After the treatment of CAR-T therapy, patients are often monitored by PET-CT instead of other treatments. As the current gold standard, PET-CT plays an important role for initial evaluation, staging, and response assessment of lymphoma 9, but PET-CT cannot significantly improve the survival 10. Besides, the high cost and risk of ionizing radiation also limit the use of PET-CT in follow-up surveillance of DLBCL 11. Alternatively, the NGS technology can overcome such limitations, and provide swift and cost-efficient analysis for follow-up surveillance.

CtDNA, released from apoptosis and/or necrosis tumor cells, is an emerging biomarker for lymphoma 12-15. The convenience of extracting ctDNA from blood has facilitated the identification and serial monitoring of tumor mutations. Compared to the tissue biopsy, plasma ctDNA has shown several advantages, such as accessibility to the difficult tumor sites, and representation of the tumor heterogeneity 16. The application of ctDNA surveillance includes MRD monitoring using immunoglobulin high-throughput sequencing 12, 13, which can also track the MRD of patients undergoing CAR T-cell therapy 17. However, this method is only targeting immunoglobulin while other important genetic variations are missed. Therefore, it is necessary to explore alternative approaches for better coverage and prognostic biomarkers alongside CAR-T cell therapy.

Thus, in this study, we conducted serial ctDNA monitoring and analysis using a cancer-related gene NGS panel for 8 DLBCL patients who underwent CAR-T cell therapy. The results provide insights into tumor evolution, prognosis and precision medicine for the DLBCL patients after CAR-T cell therapy.

## Methods and Materials

### Sample collection and DNA extraction

In this study, we retrospectively collected clinical data, tumor sample preserved in formalin fixed paraffin-embedded (FFPE) tissue or liquid nitrogen storage and serum preserved in liquid nitrogen storage from the prospective clinical study (NCT03118180). We used the panel-based NGS approach to profile mutation landscapes ofthe baseline, relapsed, and progressive tumor sample after CAR-T therapy. 38 plasma samples and 9 tissue from 8 DLBCL patients received CAR-T therapy between December 2016 and November 2017 were collected for this study.

The NGS tests were performed in a centralized clinical testing center (Nanjing Geneseeq Technology Inc.), according to protocols reviewed and approved by the ethical committee of the First Affiliated Hospital, Zhejiang University School of Medicine. 8-10ml of peripheral blood was collected in EDTA-coated tubes (BD) and centrifuged at 1,800g for 10 min within 2h of collection to separate the plasma and white blood cells. 5-10 ml pleural effusion, ascites or cerebrospinal fluid was centrifuged at 2,500g for 15 min to separate supernatants from floating cells. The supernatant was isolated for the extraction of cell-free DNA (cfDNA) and cell pellets were used for genomic DNA extraction. cfDNA from plasma and the supernatants of body fluids was extracted using the QIAamp Circulating Nucleic Acid Kit (QIAGEN). Genomic DNA from the cell samples was extracted using the DNeasy Blood & Tissue Kit (QIAGEN), while FFPE genomic DNA was purified using the QIAamp DNA FFPE Tissue Kit (Qiagen). Oral swab DNA was prepared by QIAamp DNA Mini Kit (QIAGEN) as control for germline mutations. All DNA was quantified by Qubit 3.0 using the dsDNA HS Assay Kit (Life Technologies), and the quality was evaluated by a Nanodrop 2000 (Thermo Fisher).

### Library preparation and sequencing

cfDNA or fragmented genomic DNA (300~350bp with Covaris M220 instrument) underwent sequencing library preparation using KAPA Hyper Prep kit (KAPA Biosystems). In brief, DNA was experienced with end-repairing, A-tailing, adapter ligation, size selection using Agencourt AMPure XP beads (Beckman Coulter), and then was amplified by polymerase chain reaction (PCR) and purified before targeted enrichment.

Indexed DNA libraries were pooled up to 2 µg together with Human cot-1 DNA (Life Technologies) and xGen Universal blocking oligos (Integrated DNA Technologies) as blocking reagents. Customized xGen lockdown probes panel (Integrated DNA Technologies) covering 413 predefined cancer-related genes was used to perform hybridization capture. Enriched libraries were sequenced on Hiseq 4000 NGS platforms (Illumina) to targeted mean coverage depths of at least 100x for swab control samples, 500x for tumor or cell genomic DNA and 3000x for cfDNAs.

### Data processing and analysis

Sequencing data were demultiplexed by bcl2fastq (v2.19), analyzed by Trimmomatic to remove low-quality (quality<15) or N bases.^18^ Then the data were aligned to the hg19 reference human genome with the Burrows-Wheeler Aligner (bwa-mem) 19 and further processed using the Picard suite (available at: https://broadinstitute.github.io/picard/) and the Genome Analysis Toolkit (GATK).^20^ SNPs and indels were called by VarScan2 21 and HaplotypeCaller/UnifiedGenotyper in GATK. Common SNPs were removed using dbSNP and the 1000 Genome data sets. Germline mutations were filtered out by comparing to the oral swab controls. A mutation was called when the mutant allele frequency (MAF) cutoff was ≥ 0.5% for tissue samples, 0.3% for liquid biopsy samples, and a minimum of three unique mutant reads on different strands with good quality scores and manually inspected in Integrative Genomics Viewer Software (IGV, Broad Institute). Gene fusions were identified by FACTERA^22^ and copy number variations (CNVs) were analyzed with ADTEx.^23^

### Statistical analysis

Overall survival (OS) was defined as the time from CAR-T cell infusion to death. Patients who did not experience an event were censored at the date of the final follow-up. Kaplan-Meier (KM) curves for OS were generated, and the log-rank test was used to compare differences between subgroups. The median follow-up time was estimated using reverse Kaplan-Meier curves.^24^ The results of PET-CT scan was the current gold standard for evaluating response to therapy in lymphoma. Sensitivity is equal to the rate of both detection methods being positive and PETCT being positive. Specificity is equal to the rate that both detection methods are negative and PETCT is negative. All quoted P values are two-tailed, with values less than 0.05 considered to be statistically significant. All calculations were performed using R software (version 4.0.3).

## Results

### Patient overview

Eight patients with r/r DLBCL were treated with CAR-T cell therapy targeting CD19. The patient characteristics were summarized in **Table 1**. The median age and the average number of prior lines of therapy were 36.5 years (range, 27 to 60), and 3.5 (range, 2 to 7), respectively. Patient 1 and Patient 5 received autologous stem cell transplantation (ASCT).

**Figure 1:**
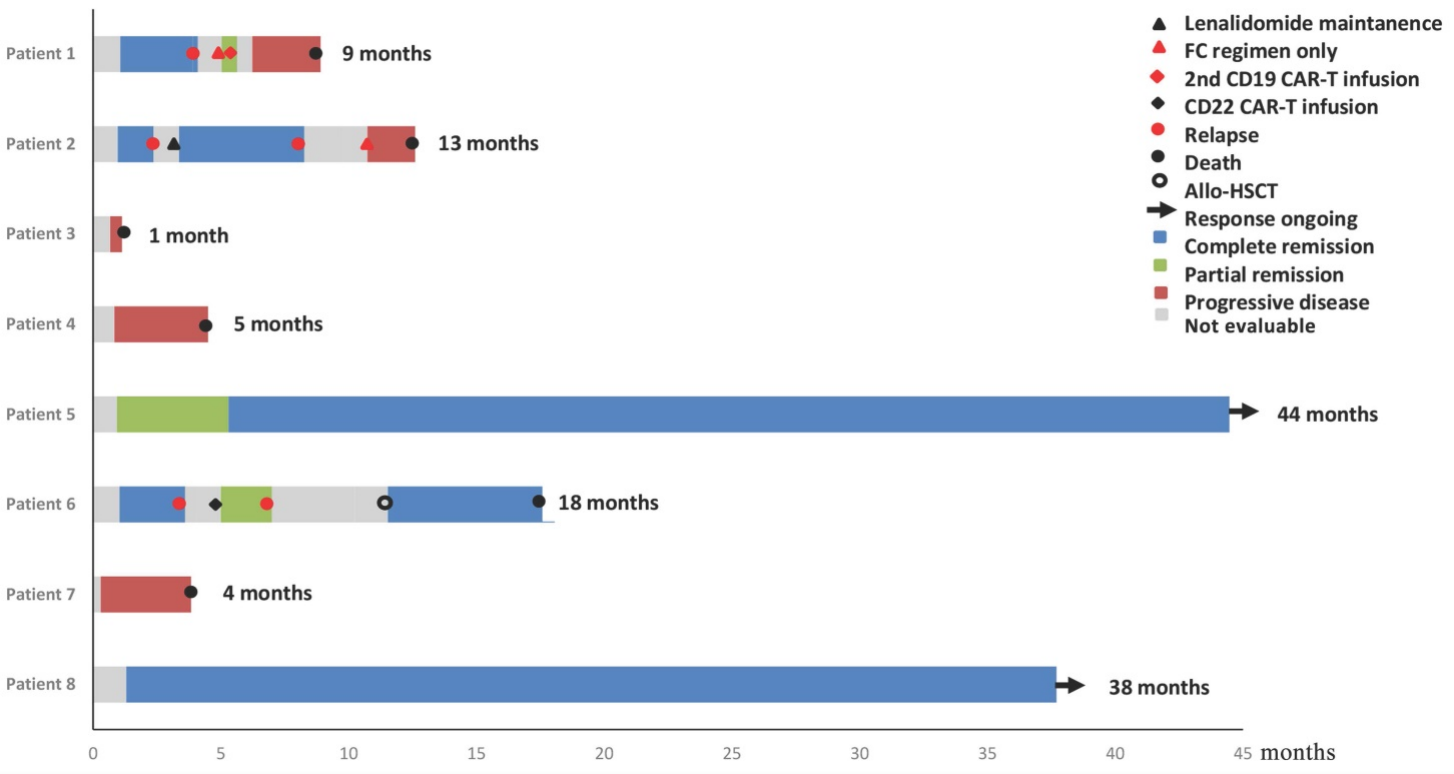
Duration of response to CD19 CAR-T and post-infusion survival in 8 cases.

**Figure 1** showed the overall survival (median: 41 months), the response of individuals, and the duration of follow-up for patients. Briefly, four patients (50%) achieved CR, one patient (12.5%) achieved partial remission (PR) and the rest three (37.5%) showed no response at 1 month after infusion of CAR-T cell. Three of the five patients who achieved remission (2 CR and 1 PR) relapsed within 4 months after CAR-T cell therapy, while the other 2 patients remained CR for more than 36.4 months and 39.2 months, respectively. Six patients experienced cytokine release syndrome (CRS), whereas Grade 1, 2, and 3 CRS occurred in two, one, and three patients, respectively.

### Serial monitoring of ctDNA mutational status

**Figure 2:**
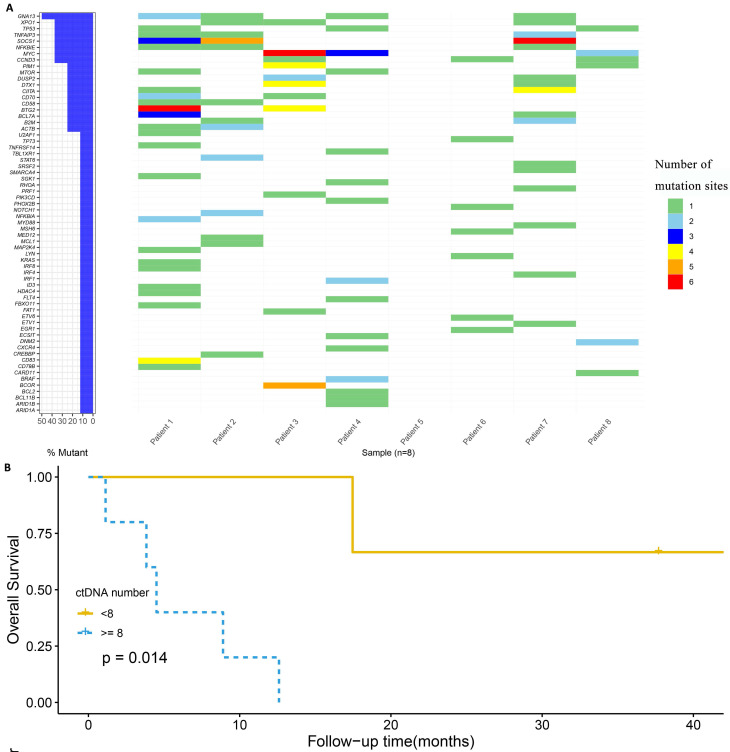
Correlation of ctDNA with prognosis. A: Mutational profile in baseline plasma ctDNA of the 8 DLBCL patients with CAR-T therapy; B: Overall survival among patients with different number of ctDNA mutation.

We performed targeted NGS detection of 89 and 446 cancer hotspot genes for Patient 8 and the other 7 patients, respectively. The DNA sequencing of 9 tissue samples were basically consistent with the ctDNA results in plasma at the same time point. The median number of baseline ctDNA mutation is 3 and 14.3 in the patients who remained long-term CR and the ones who achieved relapse/refractory after CAR-T therapy, respectively (**Figure 2A**). Due to the limited size of this study, we manually inspected the relationship between ctDNA mutation and patient's prognostic status, and observed mutations in specific genes that are likely to associate with prognosis. In particular, *GNA13* mutation was observed in 4 of 6 patients (66.7%) relapsed or refractory after CAR-T cell therapy. *SOCS1, TNFAIP3* and *XPO1* mutations occurred 3 times in the 6 relapse/refractory patients. Additionally, *DTX1*, *DUSP2*, *EGR1*, *GNA13* and *XPO1* mutations were observed in 2 of the 3 patients who never had CR. Compared with patients carrying ≥ 8 baseline serum ctDNA mutations, patients carrying < 8 mutations appear to be associated with better survival (*p* = 0.014,** Figure 2B**).

**Figure 3:**
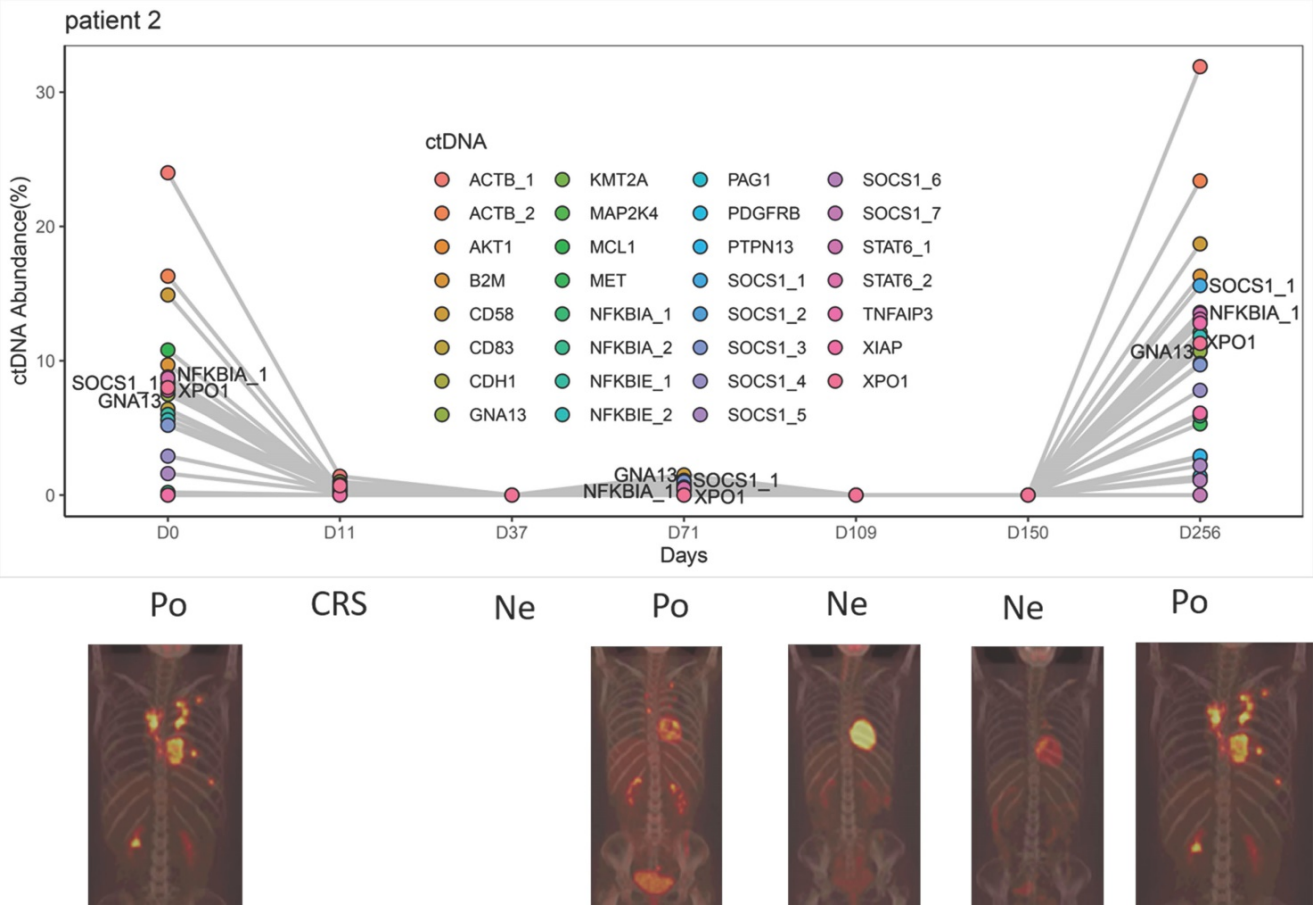
Clinical and ctDNA course of patient 2. Po: positive PET-CT; Ne: negative PET-CT; CRS: cytokine release syndrome.

As shown in **Figure 3** and **Figures S1-S7**, the trend of ctDNA surveillance exhibited good agreement with the PET-CT results. When PET-CT was positive, 18 of 19 samples (94.7%) showed gene mutations in ctDNA. Meanwhile, 10 of the 12 PET-CT negative samples (83.3%) appeared to be ctDNA mutation-free based on our targeted sequencing.

In general, we did not detect ctDNA mutation in patients with CR status, while the abundance of ctDNA mutations in the progressive disease (PD) or relapsed patients exhibited an increase. Patients at the PR status carried ctDNA mutations, but the mutation abundance was much lower than their baseline level. Of note, the change in mutation abundance was even more drastic in the aforementioned frequently mutated genes such as* GNA13, SOCS1, XPO1* and* TNFAIP3*.

### Clinical and ctDNA surveillance of Patient 2

Patient 2, diagnosed as stage IVB non-germinal center B cell DLBCL, had received 4 prior lines of therapy before CAR-T treatment. Before the CAR-T cell therapy, she had baseline ctDNA mutations in 14 genes, including *GNA13*, *SOCS1, NFKBIA* and *XPO1* (**Figure 3**). The abundance of ctDNA mutations decreased drastically at the point of CRS on Day 11, and no ctDNA mutation was detected at the CR status on Day 37. On Day 71, lymphoma recurred alongside the detection of ctDNA mutations. After the application of lenalidomide therapy, Patient 2 reached CR again and maintained CR for approximately 6 months, while no ctDNA mutation was detected during this period (Day 109 and Day 150). Afterward, Patient 2 relapsed again, underwent 3 lines of chemotherapy and died of PD. Meanwhile, we detected a strong rebound of ctDNA mutation abundance during PD (Day 205).

We proposed that ctDNA sequencing could guide precision medicine for DLBCL patients. In particular, to understand how lenalidomide contributed to relieving recurrent lymphoma here, we conducted KEGG enrichment analysis using the mutant genes at relapse detected on Day 71 (**Figure 3**). The results showed the enrichment of the term “NF-kappa B signaling pathway”. Furthermore, there were stop codon mutations *NFKBIA* 202C>T (p.Q68*) and *NFKBIE* 433A>T (p.K145*) present in ctDNA, which may result in activation of NF-Kappa-B signaling by reducing its inhibitors *NFKBIA* and* NFKNIE* expression 25. This may explain why lenalidomide relieves the subsequent relief of lymphoma recurrence in this case.

## Discussion

To our knowledge, this is the first study of serial ctDNA monitoring in r/r DLBCL patients with CD19-targeted CAR-T cell therapy. Here, we found baseline plasma ctDNA may be used as prognostic markers for r/r DLBCL patients. Patients carrying ctDNA mutations in the genes of *GNA13, SOCS1, TNFAIP3, DTX1, DUSP2, EGR1* and* XPO1*, or having over 7 mutated genes in the baseline plasma ctDNA simultaneously may be associated with poor prognosis. Our ctDNA mutation profiling results of the r/r patients with CAR-T therapy were consistent with the previous study on the mutation landscape of DLBCL patients 26. We also confirmed that serial plasma ctDNA analysis could be used as an approach for identifying the burden of disease and further monitoring disease prognosis after CAR-T cell therapy. This finding was in accordance with the result of DLBCL patients receiving traditional chemotherapy 26. The targeted NGS method with cancer-related gene panel used in this study demonstrates advantages over other traditionally DLBCL monitoring methods. First of all, the detection ability limits the application of radiological imaging tools for DLBCL. For example, the sensitivity of CT is low for accurately detecting DLBCL. Furthermore, radiation exposure and invasiveness of CT and PET-CT also restrict the frequency of using these imaging methods for serial monitoring of DLBCL status 13. Based on the current gold standard of PET-CT scan, the sensitivity and specificity of ctDNA profiling in our experience for detecting ctDNA mutation is 94.7% and 83.3%, respectively. The convenience of ctDNA sequencing would also enable multi-point surveillance during the interval between PET-CT examinations. Alternatively, checking ctDNA encoding VDJ genes can detect disease recurrence at an early stage with quantitative characteristics 12, 13. However, this method conveys relatively limited information about the mutation spectrum, clonal evolution and resistance mechanism 11, while our approach of sequencing the predefined gene panel can overcome these limitations and further facilitate precise treatment of DLBCL.

CAR-T cell therapy is usually the last resort for the treatment of r/r DLBCL patients after traditional therapies have failed, therefore, it is essential to keep exploring new options for patients who relapse or become refractory after CAR-T treatment. CRS is the most important side effect after CAR-T reinfusion and the CRS grade is closely related to many indicators after CAR-T treatment. However, no significant conclusions on CRS and ctDNA were detected in this research. Previous studies have shown that serial NGS-based ctDNA tests can be used to analyze the clonal evolution of disease progression, reveal the transformation of dominant subclones, and significantly benefit disease treatment 27-29. Here, we explored the longitudinal ctDNA change after CAR-T therapy, the results provide novel insights into precise treatment, and potentially benefit patients experiencing progression or relapse after CAR-T infusion. For instance, we reported our experience of lenalidomide in the treatment of relapse for Patient 2. After lymphoma recurrence, the patient received lenalidomide therapy, then reached CR and maintained CR for about 6 months before relapse again. Baseline plasma ctDNA sequencing revealed that this patient carried nonsense mutations in *NFKBIA* 202C>T (p.Q68*) and *NFKBIE* 433A>T (p.K145*), which may result in the decrease of their gene products due to haploinsufficiency. *NFKBIA* and* NFKBIE* encode the cytosolic NF-κB inhibitors IκBα and IκBɛ, whereas inactivating the inhibitors can result in activation of NF-Kappa-B signaling, which is a hallmark of DLBCL 25. Due to its activity against NF-Kappa-B signaling, lenalidomide therapy may synergize with CAR-T cell therapy and facilitate the antitumor function of CAR-T therapy 30. This could explain why lenalidomide relieved the recurrence of lymphoma in this case and may become a good choice for DLBCL treatment. Another candidate that holds promise is* XPO1* (Exportin 1). We discovered recurrent *XPO1* 1711G>A (p.E571K) mutation in 3 out of 6 relapsed/refractory patients. Exportin 1 contributes to cell homeostasis by regulating the export of protein and RNA molecules from the nucleus to the cytoplasm 31. As many tumor suppressors and oncoproteins use Exportin 1 as their mechanism for nuclear export, cancer cells can utilize this nuclear-cytoplasmic transport process to evade anti-neoplastic mechanisms 31, 32. Moreover, the E571K mutation of *XPO1* is highly prevalent in several cancers, and likely affects nuclear exporting by altering the nuclear export signal (NES)-binding groove of Exportin 1 33. Thus, this mutation could be related to the drug resistance of different treatment methods (including chemotherapy and targeted therapy), making it an attractive target for new cancer therapies 34. Notably, the *XPO1* inhibitor selinexor can be used for the treatment of multiple myeloma and DLBCL, and the FDA has approved selinexor for adult patients with r/r DLBCL 35-37. So, selinexor can be another therapy choice. Due to the small sample size of this retrospective study, future validation of lenalidomide and selinexor treatments is warranted.

Taken together, our work has demonstrated the use of serial ctDNA surveillance for r/r DLBCL patients after CD19-targeted CAR-T cell therapy, which can provide important insights into the prognosis of individual patients. Serial plasma ctDNA monitoring can identify genetic variants in inaccessible tumor sites and better represent the heterogeneity of the entire tumor. Thus, not only can it be used to monitor MRD, it also shows great potential to reveal prognosis, stratify patients and guide precision treatment after CAR-T cell therapy. As this is a single-center retrospective study of 8 patients, our findings need to be verified on a large scale. Besides, we only collected plasma samples for ctDNA mutation surveillance during the limited number of PET-CT follow-up, while plasma ctDNA mutations can be monitored more frequently between PET-CT examinations in the future.

## Conclusion

In summary, here we report the first study on panel-based NGS for serial ctDNA monitoring of r/r DLBCL patients undergoing CAR-T cell therapy. Our study indicates that the dynamic change of tumor ctDNA could potentially serve as the biomarker for predicting and monitoring the prognosis, as well as the guidance for treatment decision-making.

## Supplementary Material

Supplementary figures.

## Figures and Tables

**Figure 4:**
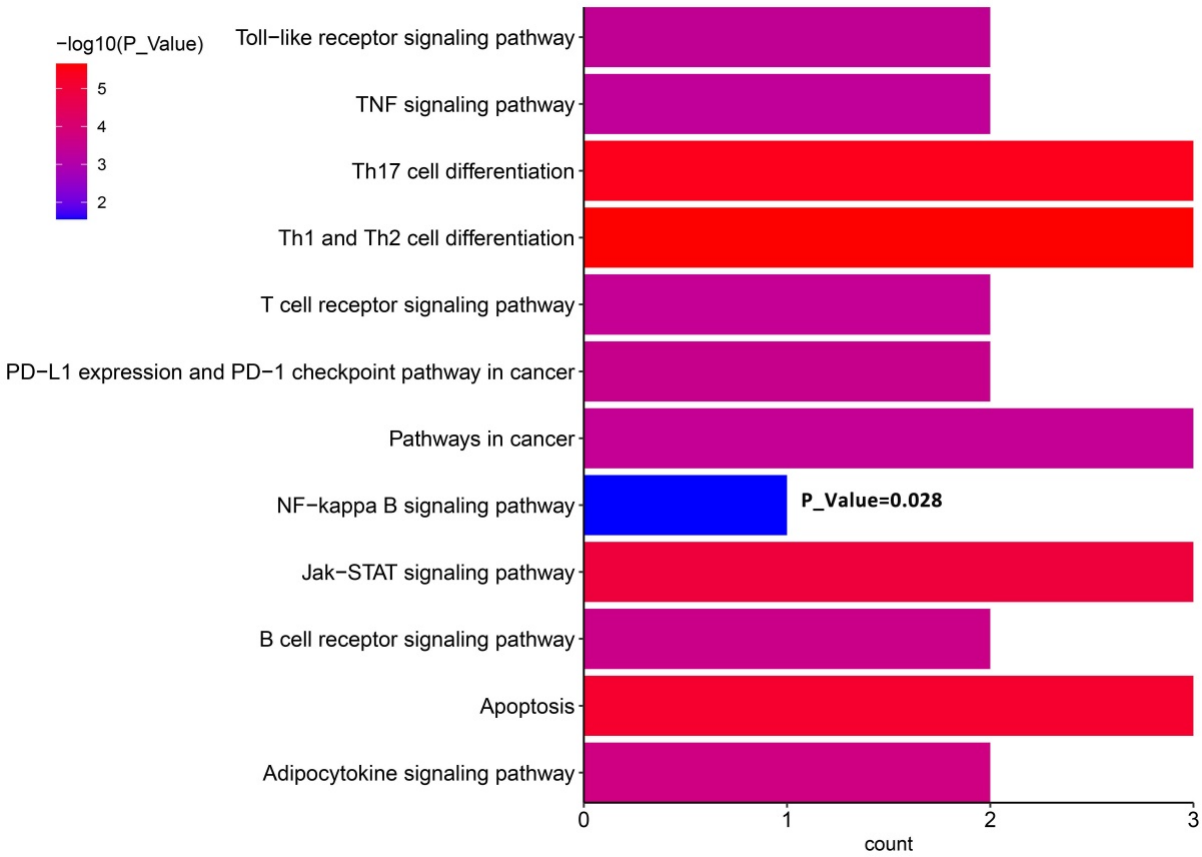
KEGG pathway enrichment analyses of ctDNA in Patient 2.

**Table 1:** Clinical Characteristics of patients

Patient	1	2	3	4	5	6	7	8
Age (years)	35	27	36	44	43	60	33	50
Sex	Male	Female	Male	Female	Female	Female	Female	Male
Diagnosis	DLBCL	DLBCL	DLBCL	DLBCL	DLBCL	DLBCL	DLBCL	DLBCL
Cell of origin	non-GCB	non-GCB	non-GCB	NOS-GCB	non-GCB	ABC	NA	non-GCB
NCCN-IPI	3	3	3	3	3	2	2	2
Risk group	Low-intermediate	Low-intermediate	Low-intermediate	Low-intermediate	Low-intermediate	Low-intermediate	Low-intermediate	Low-intermediate
Prior lines of therapy	7	4	3	3	5	2	3	4
Prior therapies	(1)EPOCH (2)RCHOP (3)RCHOPE (4)RIVAC (5)RAD (6)BAC-ASCT (7)Thalidomide+rituximab	(1)R-DA-EPOCH (2)RCHOP (3)RCHOPE (4)RGDP	(1)IEP (2)RCHOP (3)GDP	(1)RCHOP (2)R (3)DHAP	(1)RCHOP (2)RCHOPE (3)R (4)ESHAP (5)ASCT	(1)RCHOP (2)R2+ ibrutinib	(1)RCHOP (2)RESHAP (3)GDP	(1)CHOP (2)RCHOP (3)REPOCH (4)CHOP+ Lenalidomide
Relapse/ refractory status	First relapsed post-EPOCH within 11 years Second relapsed post-ASCT within 3 months	Refractory second or higher line of therapy	Refractory second or higher line of therapy	Refractory second or higher line of therapy	First relapsed post-R within 4 months Second relapsed post-ASCT within 6 months	Refractory second or higher line of therapy	Refractory second or higher line of therapy	Refractory second or higher line of therapy

ABC, activated B-cell-like; AD, albumin-bound paclitaxel, liposomal doxorubicin; ASCT, autologous stem cell transplant; BAC, carmustine, cytarabine, cyclophosphamide; CHOP, cyclophosphamide, adriamycin, vincristine, prednisone; CHOPE, rituximab, cyclophosphamide, adriamycin, vincristine, prednisone, etoposide; DA, dose adjusted; DHAP, dexamethasone, high-dose cytarabine, cisplatin; DLBCL, diffuse large B cell lymphoma; EPOCH, etoposide, prednisone, vincristine, cyclophosphamide, doxorubicin; ESHAP, etoposide, methylprednisolone, high-dose cytarabine, cisplatin; GCB, germinal center B cell; GDP, gemcitabine, dexamethasone, cisplatin; ICE, ifosfamide, carboplatin, etoposide; IEP, Ifosfamide, epirubicin, cisplatin; IVAC, rituximab, ifosfamide, cytarabine, etoposide; NCCN-IPI, National Comprehensive Cancer Network-International Prognostic Index; R, rituximab; R2, rituximab, lenalidomide.

## Abbreviations

CtDNA: circulating tumor DNA
NGS: next-generation sequencing
MRD: minimal residual disease
DLBCL: diffuse large B cell lymphoma
CAR-T: chimeric antigen receptor T
CR: complete remission
PR: partial remission
PD: progressive disease
ASCT: autologous stem cell transplantation
FFPE: formalin fixed paraffin-embedded
