# Supplementary material for: Serial surveillance by circulating tumor DNA profiling after chimeric antigen receptor T therapy for the guidance of r/r diffuse large B cell lymphoma precise treatment
Source: J Cancer. 2021 Jul 13;12(18):5423–31. doi: 10.7150/jca.60390 (PMC8364638; doi:10.7150/jca.60390)

Surveillance by circulating tumor DNA profiling after CAR-T therapy for the guidance of r/r DLBCL precise treatment

Linghui Zhou[#] [1,2,3,4], Houli Zhao[#] [1,2,3,4], Wei Ding[5], Yang Shao[6], Xin Chen[6], Ruimin Hong[1,2,3,4], Linqin Wang[1,2,3,4], Fang Ni[1,2,3,4], Arnon Nagler[7], Yongxian Hu[1,2,3,4], He Huang[1,2,3,4]

1.Bone Marrow Transplantation Center, the First Affiliated Hospital, Zhejiang University School of Medicine

2. Institute of Hematology, Zhejiang University

3. Zhejiang Province Engineering Laboratory for Stem Cell and Immunity Therapy

4.Liangzhu Laboratory, Zhejiang University Medical Center, 1369 West Wenyi Road, Hangzhou 311121, China

5. Department of Pathology, the First Affiliated Hospital, School of Medicine, Zhejiang University, Hangzhou, China;

6. Nanjing Geneseeq Technology Inc., Nanjing, Jiangsu, China;

7. Chaim Sheba Medical Center, Tel Hashomer, Israel, Tel Hashomer, Israel.

# Contributed equally.

Corresponding author:

Yongxian Hu, MD/PhD

Bone Marrow Transplantation Center, The First Affiliated Hospital, School of Medicine, Zhejiang University. No.79 Qingchun Road, Hangzhou, China.

Telephone and Fax: 86-0571-87236706 E-mail: 1313016@zju.edu.cn

He Huang, Professor/MD/PhD

Bone Marrow Transplantation Center, The First Affiliated Hospital, School of Medicine, Zhejiang University. No.79 Qingchun Road, Hangzhou, China.

Telephone and Fax: 86-0571-87236706 E-mail: huanghe@zju.edu.cn

Supp Figures

Supp Figure 1 Clinical and ctDNA course of patient 1. Po: positive PET-CT; Ne: negative PET-CT; CRS: cytokine release syndrome.

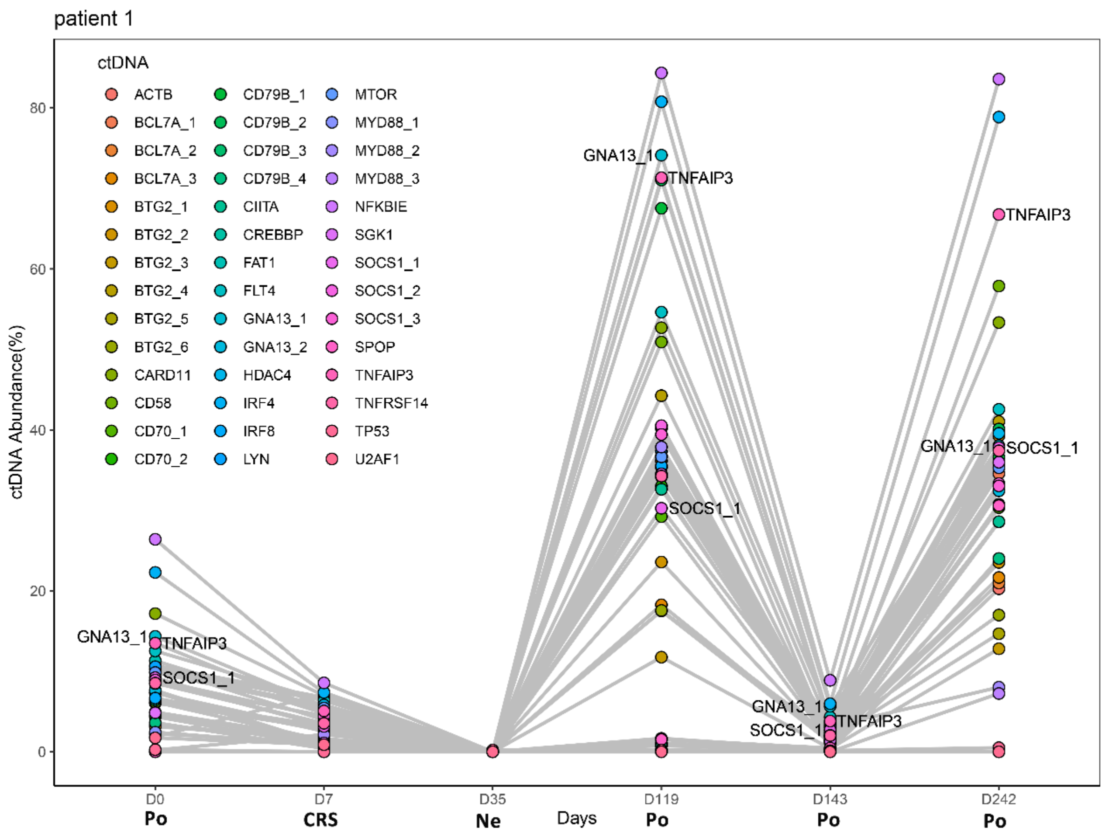

Supp Figure 2 Clinical and ctDNA course of patient 3. Po: positive PET-CT; Ne: negative PET-CT; CRS: cytokine release syndrome.

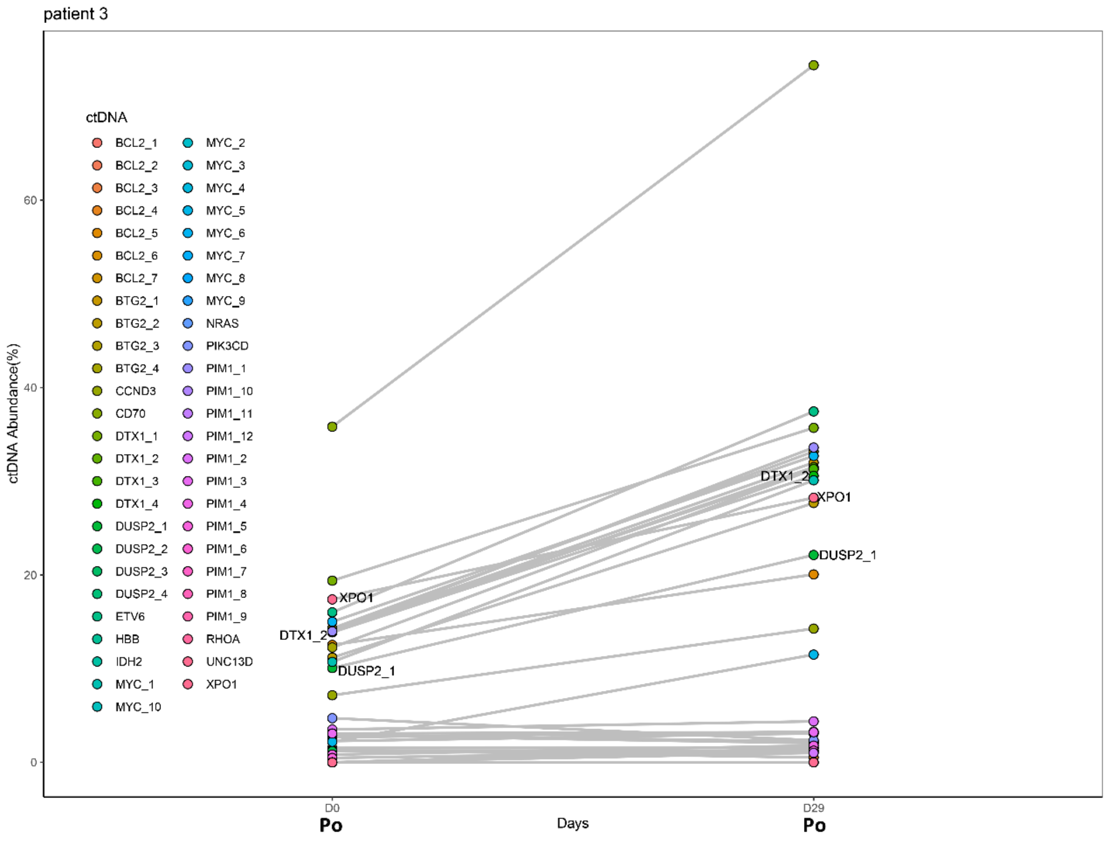

Supp Figure 3 Clinical and ctDNA course of patient 4. Po: positive PET-CT; Ne: negative PET-CT; CRS: cytokine release syndrome.

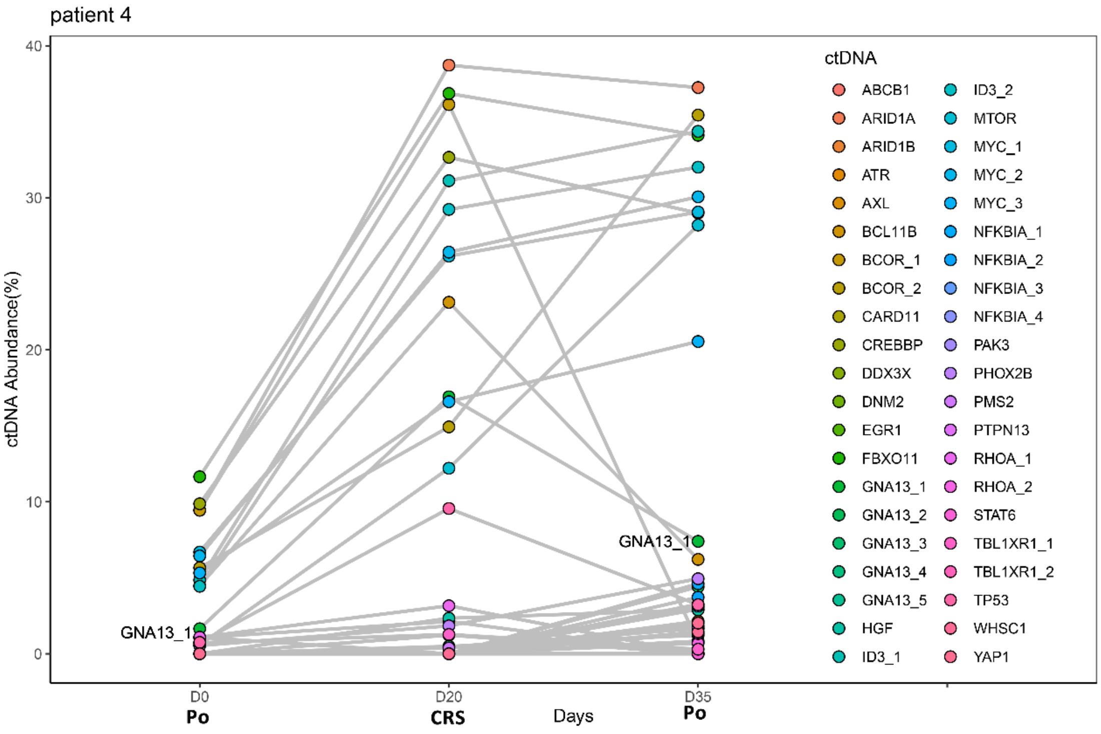

Supp Figure 4 Clinical and ctDNA course of patient 5. Po: positive PET-CT; Ne: negative PET-CT; CRS: cytokine release syndrome.

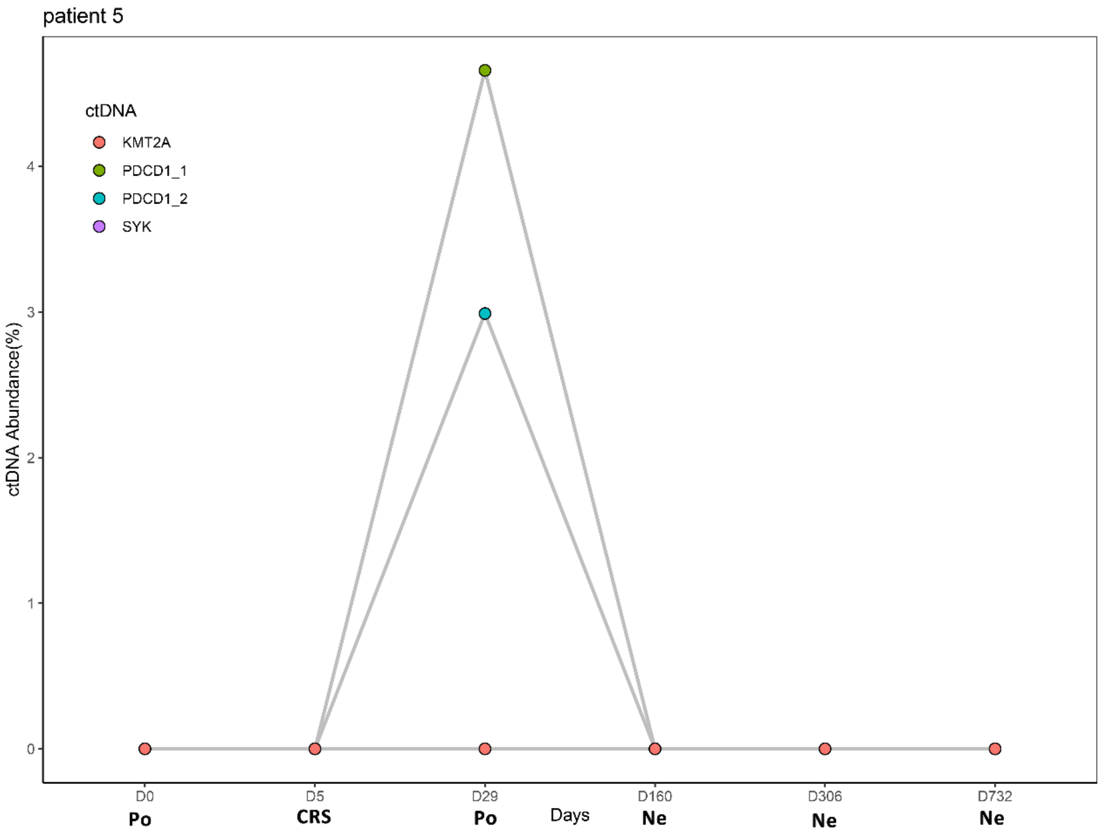

Supp Figure 5 Clinical and ctDNA course of patient 6. Po: positive PET-CT; Ne: negative PET-CT; CRS: cytokine release syndrome.

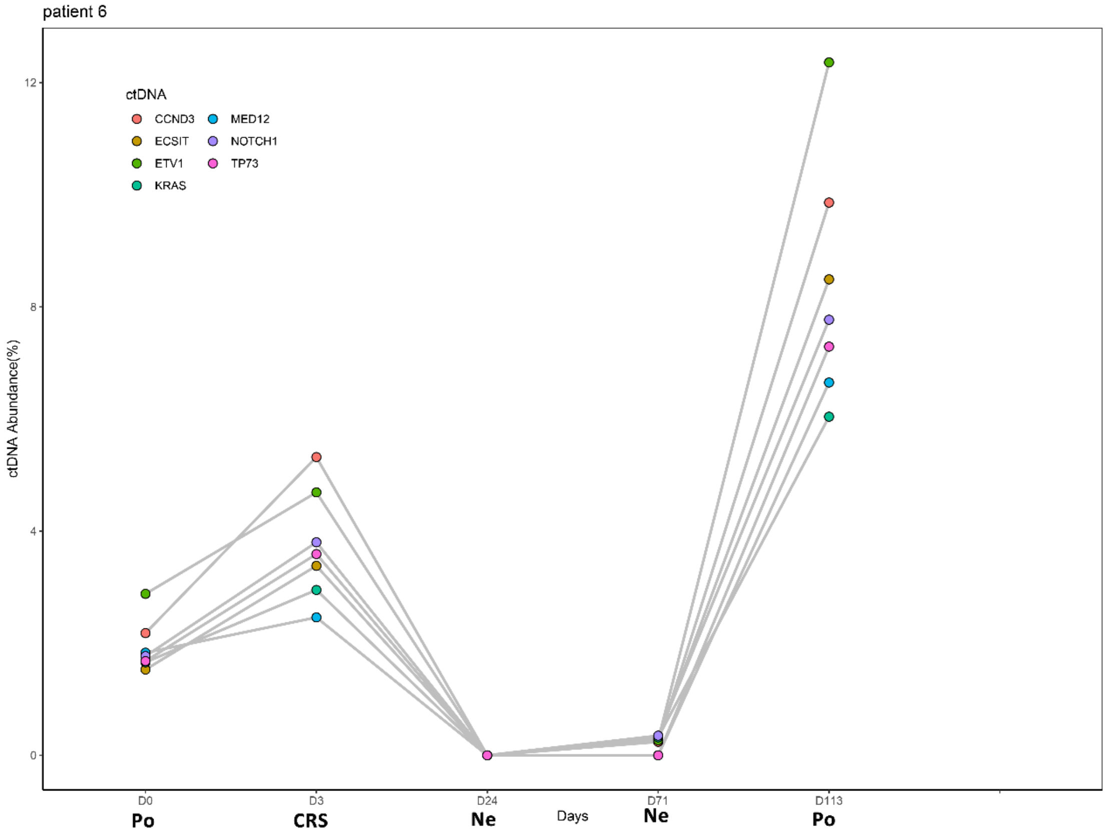

Supp Figure 6 Clinical and ctDNA course of patient 7. Po: positive PET-CT; Ne: negative PET-CT; CRS: cytokine release syndrome.

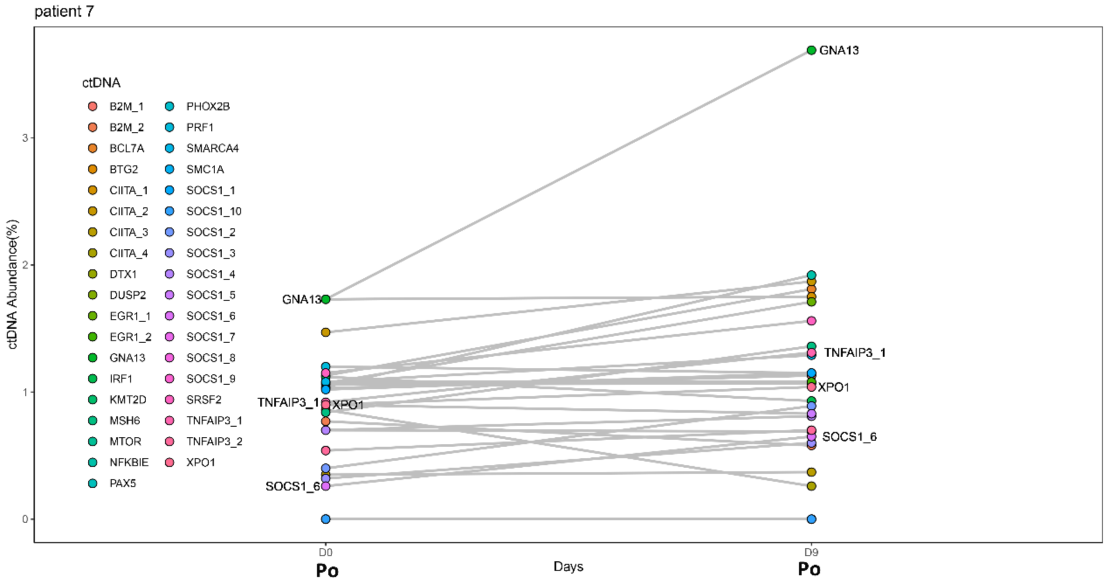

Supp Figure 7 Clinical and ctDNA course of patient 8. Po: positive PET-CT; Ne: negative PET-CT; CRS: cytokine release syndrome. The lump is obviously reduced in the ninth day.

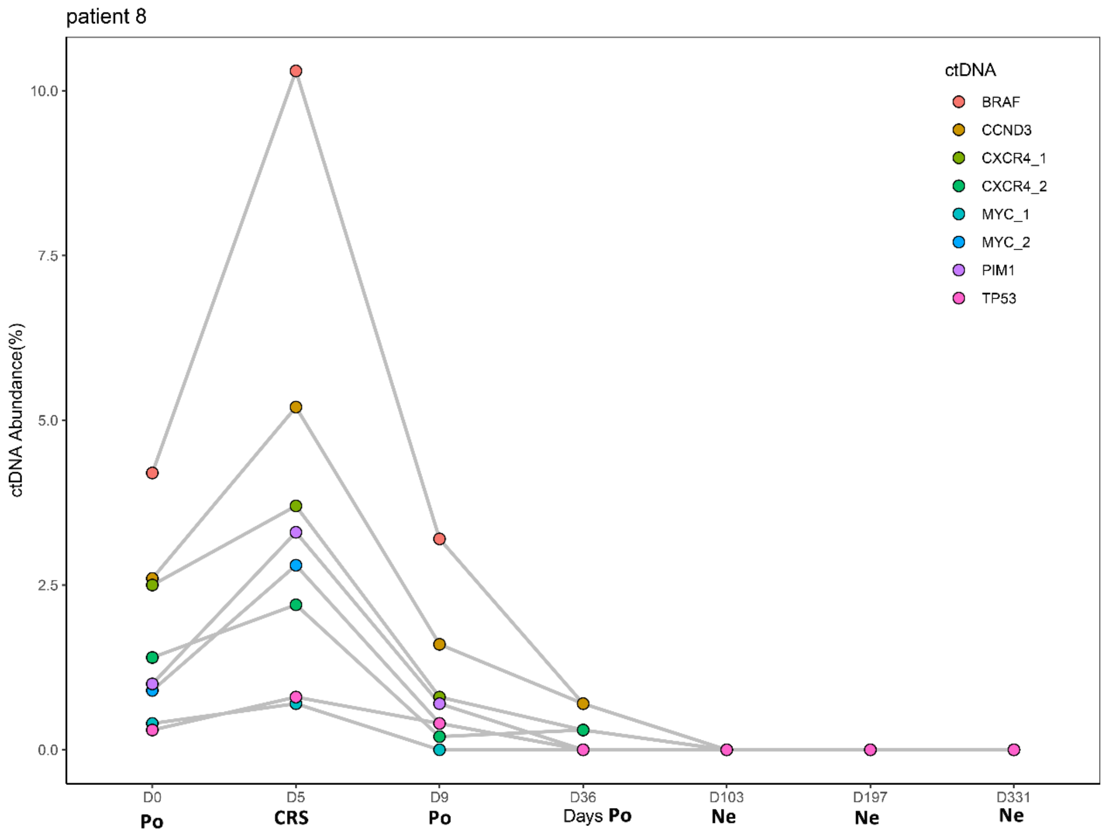

Supplement: Supplementary file 1 — Supplementary figures. [file jcav12p5423s1.pdf]
